# Using Individual Collector Blood Culture Volume Data To Improve Fill Volume

**DOI:** 10.1128/spectrum.02106-23

**Published:** 2023-07-13

**Authors:** Richard S. Kirby, Jenna M. Meloni, Karishma B. Naik, Matthew D. Minnear, Matthew A. Pettengill

**Affiliations:** a Sidney Kimmel Medical College, Thomas Jefferson University, Philadelphia, Pennsylvania, USA; b Department of Pathology and Genomic Medicine, Thomas Jefferson University, Philadelphia, Pennsylvania, USA; c Children’s Hospital of Philadelphia, Department of Pathology and Laboratory Medicine, Philadelphia, Pennsylvania, USA; University of Arizona/Banner Health

**Keywords:** blood culture, bloodstream infections, volume

## LETTER

Blood culture is the standard diagnostic modality for detection of bloodstream infections, and the sensitivity of blood culture detection is directly predicated on the blood culture fill volume (1–3). Previously published studies of quality improvement projects have demonstrated that targeted education can improve blood culture fill volumes (4–6).

As part of a quality improvement (QI) project, in addition to other educational interventions, individual collector data were obtained for analysis and distribution to collector managers. Our lab uses BD Bactec FX blood culture instruments and BD EpiCenter middleware. We were able to access individual collector data in EpiCenter using the Reports tab in Bactec FX Instrument reports and accessing the Blood Volume Monitoring Reports folder. Then, we selected Blood Volume Summary, set the study period for Collection Date Selection, and under Group Selection we checked Collected By, where we displayed a list of collectors' ID numbers, and we selected all ID numbers and then selected Print Preview and exported the data as a spreadsheet. In our case, the individual collector ID numbers were converted to employee names and direct reports by our EPIC lab information system group. Only collectors (or locations) with at least 25 collections per study period were included in the report.

**FIG 1:**
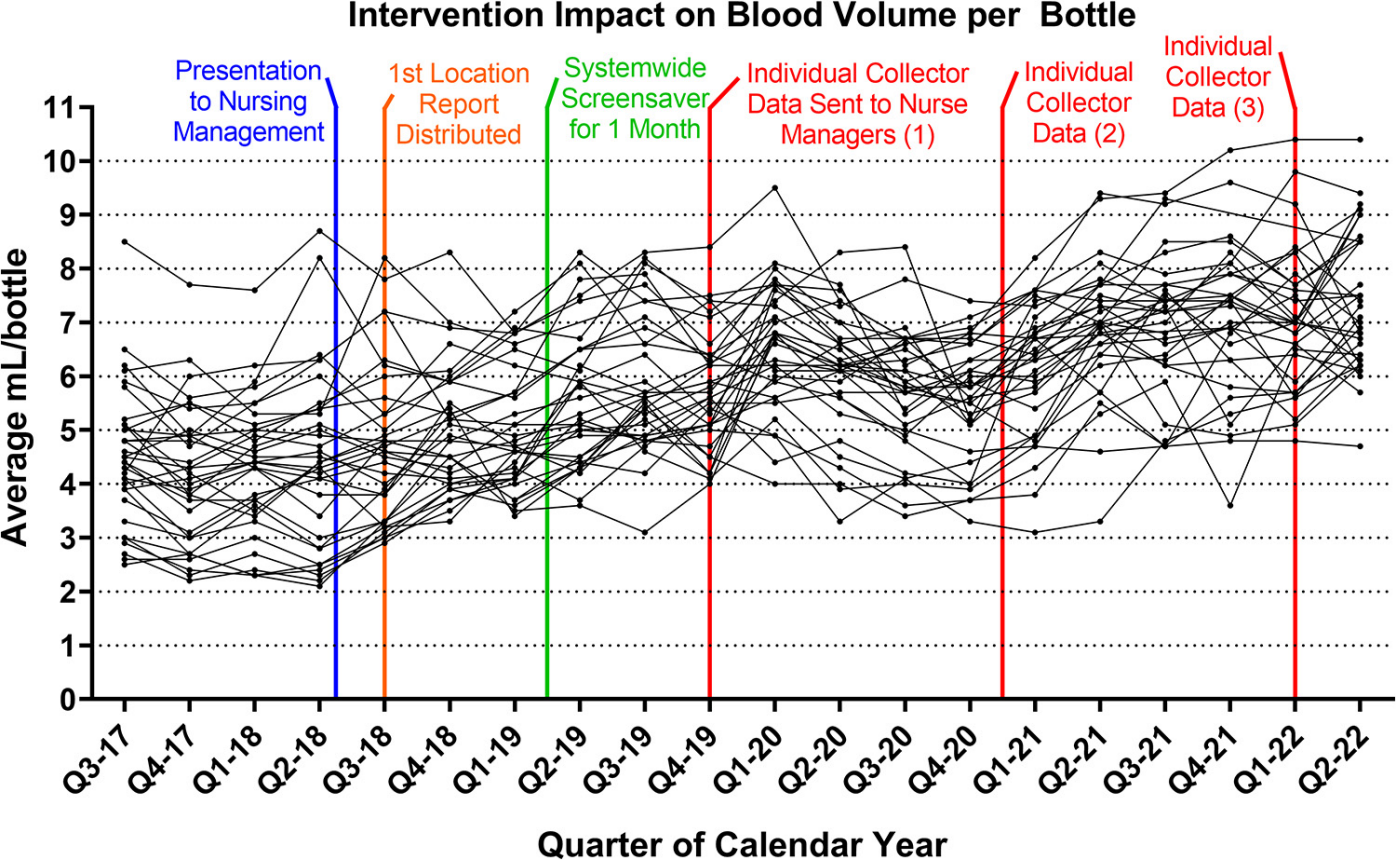
Average blood culture fill volume trend over the course of the QI project. Each line is one specific hospital ward.

Individual collector data were used both for creating reports for nurse managers and phlebotomy supervisors and to study a 2-year period of individual collectors, correlating their average volume and the individual collector positive blood culture rate. The latter activity was approved by our institutional review board (IRB control number 20E.112). For the 2-year study period (calendar years 2018 to 2019), collectors who averaged 0 to 2.5, 2.6 to 5.0, 5.1 to 7.5, and >7.5 mL per blood culture had positive culture rates of 7.10%, 7.72%, 7.76%, and 8.00%, respectively (from 9,889 to 28,722 total cultures per category). In 2018, nurse managers were provided with location-specific blood culture fill volume (BCFV) data and education regarding proper BCFV levels. In fiscal year 2018 (FY18, July 2017 to June 2018), the average BCFV was 4.63 mL, with a true positive blood culture rate of 7.0%. In 2019, a health system-wide screen saver with information about appropriate BCFV was implemented for 1 month on all hospital computers. Beginning in the first quarter of 2020, nurse managers were provided with collector-specific BCFV data, which they used for team meeting presentations and individual discussions with low collectors, and this format of data was subsequently provided annually as shown in Fig. 1. In addition to the data used for corrective instruction, gold and silver blood culture bottle-shaped lapel pins were provided for collectors achieving >9.5 and 9.0 to 9.5 mL/bottle averages, respectively, which nurse managers indicated created some healthy competitive behavioral changes and provided a regular discussion opportunity to reinforce education on correct collection practices. Percent change in average blood culture volume was calculated for each quarter and compared to the previous quarter (for each pair for which sufficient data were available for a given ward in each quarter) and separated into the following groups: no intervention (no educational activities in the previous quarter), other intervention (presentation to nursing key personnel, initiation of quarterly ward-specific reports, screensavers), or collector-specific intervention (distribution of individual collector data to nursing and phlebotomy managers). For no-intervention quarters (431 measurements), there was an average increase of 0.85%. For other interventions there was an average 10.8% increase (102 measurements), and for collector-specific interventions there was as an average 11.4% increase (99 measurements). The improvement following each type of intervention was statistically significant compared to no intervention (two-tailed Student's *t* test, *P* < 0.001 for each comparison), but there was not a statistically significant difference between various intervention types. Over the course of the QI project, there was a 55.8% increase in BCFV, from 4.63 mL to 7.21 mL per fiscal year, and true blood culture positivity rate (excluding contaminants) also increased by 13.2%, which yielded a theoretical increased detection of 461 additional positive blood cultures per year compared to the beginning of the study after adjustment for the increased number of cultures submitted (Table 1). The changes in true percent positive blood cultures relative to the first year of the study (FY18) achieved statistical significance for FY21 and FY22 (Student's *t* test, *P* values in right-most column of Table 1; statistical analysis performed in GraphPad Prism v9.1.2).

**TABLE 1:** Blood culture fill volumes and culture positivity by fiscal year of the quality improvement project^*a*^

FY	Total no. of cultures	No. of true positives	No. of true positives/ 50,000	% true positives	Avg BCFV (mL)	*P* value
FY18	45,261	3,176	3,509	7.00%	4.63	
FY19	46,728	3,303	3,534	7.10%	5.57	0.7603
FY20	50,732	3,648	3,595	7.20%	6.00	0.2960
FY21	50,632	3,830	3,782	7.60%	6.77	0.0011
FY22	48,685	3,866	3,970	7.90%	7.21	<0.0001

a Positive cultures flagged as probable contaminants were excluded from true positives. Statistical analyses compared percent true positivity for each individual year, FY19 to FY22, compared to FY18.

One limitation of our study is that we were unable to get individual collector data for collectors submitting fewer than 25 collections per year, and although individuals collecting ≥25 cultures per year represented 63% of all blood culture collections, they were only 18% of all collectors submitting any cultures. The level of improvement in true percent positivity we observed is similar to what was achieved in another recent study utilizing other educational methods (4). Our study demonstrates that individual collector data can be used as an effective educational intervention to improve BCFV.
